# *Gmelina arborea* Roxb. (Family: Verbenaceae) Extract Upregulates the *β*-Cell Regeneration in STZ Induced Diabetic Rats

**DOI:** 10.1155/2016/4513871

**Published:** 2016-01-06

**Authors:** Anoja Priyadarshani Attanayake, Kamani Ayoma Perera Wijewardana Jayatilaka, Chitra Pathirana, Lakmini Kumari Boralugoda Mudduwa

**Affiliations:** ^1^Department of Biochemistry, Faculty of Medicine, University of Ruhuna, 80000 Galle, Sri Lanka; ^2^Department of Pathology, Faculty of Medicine, University of Ruhuna, 80000 Galle, Sri Lanka

## Abstract

*Gmelina arborea* Roxb. (common name: Et-demata, Family: Verbenaceae) has been used traditionally in Sri Lanka as a remedy against diabetes mellitus. The objective of the present study was to evaluate antidiabetic mechanisms of the aqueous bark extract of* G. arborea* in streptozotocin induced (STZ) diabetic male Wistar rats. Aqueous bark extract of* G. arborea* (1.00 g/kg) and glibenclamide as the standard drug (0.50 mg/kg) were administered orally using a gavage to STZ diabetic rats (65 mg/kg, ip) for 30 days. The antidiabetic mechanisms of aqueous extract of* G. arborea* (1.00 g/kg) were determined at the end of the experiment. The fasting blood glucose concentration was significantly lowered and the serum insulin and C-peptide concentrations were increased by 57% and 39% in plant extract treated rats on day 30, respectively (*p* < 0.05). The histopathology and immunohistochemistry results of the plant extract treated group showed a regenerative effect on *β*-cells of the pancreas in diabetic rats. In addition, serum lipid parameters were improved in* G. arborea* extract treated diabetic rats. The results revealed that the aqueous stem bark extract of* G. arborea* (1.00 g/kg) showed beneficial effects against diabetes mellitus through upregulating the *β*-cell regeneration and biosynthesis of insulin in diabetic rats.

## 1. Introduction

Diabetes mellitus (DM) is one of the most common metabolic disorders worldwide [1]. The global prevalence of diabetes mellitus has shown an upward trend over the past few decades. Every ten seconds a person dies from diabetes-related causes. DM therefore has become a very serious public health problem with a heavy socioeconomic burden to most of the South Asian countries including Sri Lanka [2, 3].

DM is characterized by hyperglycemia and alteration of carbohydrate, fat, and protein metabolisms associated with absolute or relative deficiency in insulin secretion or insulin action [1]. Present pharmacological therapies aim at correcting/overcoming these defects. However, studies have consistently demonstrated that patients' adherence to present therapeutic regimes has not been satisfactory. Regime complexity, occurrence of hypoglycemia, other side effects, lack of confidence in immediate or future benefits, and patients' education/beliefs are among the common reasons limiting compliance. Inadequacies in current treatment regimens have resulted in relying on complementary and alternative medicines for the management of diabetes [4, 5].

*Gmelina arborea *Roxb. (common name: Et-demata; Family: Verbenaceae) is valuable in Sri Lankan traditional medicine and a decoction of the bark of* G. arborea* is successfully employed for glycemic control and long term complications of diabetes mellitus by Ayurvedic physicians [6, 7]. Extensive research has been done for the investigations on phytochemicals,* in vivo* and* in vitro* antioxidant potentials and* in vivo* toxic effects by our research group [8, 9]. Furthermore, acute hypoglycemic and antihyperglycemic activities of the aqueous extract of* G. arborea* were studied in a graded dose range (0.25 g/kg–2.0 g/kg) in healthy (normoglycemic) and streptozotocin induced diabetic rats, respectively. Results of the acute study confirmed that the optimum effective antihyperglycemic dose of the* G. arborea* extract was 1.00 g/kg in streptozotocin induced diabetic rats [10].

In the present study, the effect of aqueous extract of* G. arborea* on selected glycemic parameters (fasting blood glucose concentration, percentage of glycated hemoglobin (HbA_1C_) and serum concentration of fructosamine) and lipid parameters (serum concentrations of total cholesterol: TC; high density lipoprotein cholesterol: HDL-C; triglyceride: TG; low density lipoprotein cholesterol: LDL-C; very low density lipoprotein cholesterol: VLDL-C) was evaluated. In addition, the antidiabetic mechanisms of the extract were determined through the estimation of serum concentration of insulin, C-peptide, and detailed assessments of histopathology and immunohistochemistry of the pancreas. Glibenclamide was used as the standard drug in the present study and it has been widely accepted as a standard drug in diabetic animal experiments associated with mild, severe hyperglycemia [11].

The objective of the study was to determine detailed antidiabetic mechanisms of the bark extract* G. arborea* and its potency to induce *β*-cell regeneration in the pancreas of streptozotocin induced (STZ) diabetic rats.

## 2. Materials and Methods

### 2.1. Chemicals

D-glucose, glibenclamide, and streptozotocin were purchased from Sigma-Aldrich Company (St. Louis, MO, USA). A UV visible spectrophotometer (Gallenkamp PLC, UK) and microplate reader (Mindray, China) were used for spectrophotometric and enzyme linked immunosorbent assay (ELISA) measurements, respectively. Olympus CX 21 (Japan) microscope was used in the assessment of histopathology and immunohistochemistry of the pancreatic tissues.

### 2.2. Plant Material

Stem bark parts of* G. arborea *were collected during May-June 2013 from the Southern Region of Sri Lanka. Botanical identity was determined by the descriptions given by Jayaweera [6] and confirmed by comparing authentic samples at the National Herbarium, Royal Botanical Gardens, Peradeniya, Sri Lanka. A voucher specimen was preserved at the Department of Biochemistry, Faculty of Medicine, University of Ruhuna, Sri Lanka (Attanayake/2011/01).

### 2.3. Preparation of the Plant Extract

The bark parts of* G. arborea *were cut into small pieces and dried at 40°C until a constant weight. Powdered plant material (50.00 g) was dissolved in 400.0 mL of distilled water and refluxed for 4 h. The mixture was strained and the final volume was adjusted to 50.0 mL. The dose of 1.00 g/kg was administered orally to streptozotocin induced diabetic rats.

### 2.4. Animals

Healthy adult male rats of Wistar strain (200 ± 25 g, body weight) were purchased from the Medical Research Institute (MRI), Sri Lanka, and used to carry out the experiments. They were housed in standard environmental conditions at the Animal House of Faculty of Medicine, University of Ruhuna, Sri Lanka (Temp. 25 ± 2°C, relative humidity 55–65%, and 12 ± 1 h light/dark cycle). Rats were fed with standard diet (MRI rat formulae, Sri Lanka) with free access to water before and during the experiment. The rats were randomized into various groups and allowed to acclimatize for a period of seven days under standard environmental conditions before the commencement of the experiments. The animals described as fasting were deprived of food but had access for water for 12 h. All protocols used in this study were approved by the Ethics Committee of Faculty of Medicine, University of Ruhuna, Sri Lanka, guided by the Council for International Organization of Medical Sciences (CIOMS) international guiding principles of biomedical research involving animals.

### 2.5. Development of Diabetes Mellitus in Wistar Rats

Streptozotocin dissolved in citrate buffer (0.1 M, pH 4.4) at a dose of 65 mg/kg was administered intraperitoneally to rats fasted for 12 h. Thereafter, rats were maintained on 5% D-glucose solution for the next 24 h. Rats were allowed to stabilize for three days thereafter and on the 4th day, blood samples were drawn from tail vein to determine the blood glucose concentration to confirm the development of diabetes mellitus. Rats with fasting blood glucose concentration of 12.0 mmol/L or above were considered as hyperglycemic and from 4th day onwards they were used in the experiments [12].

### 2.6. Blood/Serum Glycemic Parameters in Diabetic Rats

Oral glucose tolerance test was performed in all groups on the 1st, 7th, 14th, 21st, 28th, and 30th days. The rats were given an oral dose of glucose (3.00 g/kg) 30 minutes after the administration of the plant extract. Blood samples were collected just prior to the administration of the extract/drug (0) and at 1, 2, 3, and 4 h subsequently. Blood glucose concentration was measured immediately by the glucose-oxidase method using a glucose assay kit based on the Trinder reaction [13]. The acute effect was evaluated over a 4 h period using area under the oral glucose tolerance curve [14].

The percentage of HbA_1C_ and serum concentration of fructosamine was estimated in all rats using a spectrophotometric enzyme assay kit [15, 16]. The concentrations of serum insulin and C-peptide in all rats were estimated using enzyme linked immune-sorbent assay methods [17, 18].

### 2.7. Serum Lipid Parameters in Diabetic Rats

The concentrations of serum TC, HDL-C, and TG were estimated in all rats using spectrophotometric enzyme assay kits [19–21]. The concentrations of serum LDL-C, VLDL-C were calculated using the Friedewald formulae [22].

### 2.8. Assessment of Histopathology and Immunohistochemistry of the Pancreas of Diabetic Rats

Paraffin embedded tissue blocks of the pancreas were used for the detailed assessment of histopathology and immunohistochemistry. The sections of the pancreatic tissues were stained with hematoxylin and eosin for the light microscopic examination of histopathology changes of pancreatic tissue in all rats. Histopathology score was developed for the assessment of selected histological parameters of destruction of islet cells and regeneration of islet cells [23]. The criteria for scoring the islet cell destruction were as follows: score 0 (normal): normal number of islet cells; score 1 (mild): loss of 1/3 of islet cells; score 2 (moderate): loss of 1/3 to 2/3 of islet cells; score 3 (severe): loss of more than 2/3 of islet cells. The criteria for scoring the regeneration were as follows: score 0 (none): no regeneration; score 1 (mild): regeneration of 1/3 of islet cells; score 2 (moderate): regeneration of 1/3 to 2/3 of islet cells; score 3 (prominent): regeneration of more than 2/3 of islet cells. Immunohistochemical staining was done to confirm the presence of insulin secreting cells in the islets of pancreas in all rats. Dako polyclonal guinea pig anti-insulin and Dako REAL EnVision/HRP, Rabbit/Mouse, were used for immunohistochemical staining. Islets were observed on light microscopy (high power field).

Islets were defined as being small, average, and large with an islet diameter of ≤125 *μ*m, 126–149 *μ*m, and ≥150 *μ*m, respectively [24]. Four islets of each size in each rat (72 islets for each group) were chosen randomly [25]. The percentage of insulin secreting *β*-cells in islets and islet profile diameter were estimated [24, 25].

### 2.9. Statistical Analysis

Results are expressed as mean ± SEM for biochemical estimations. The quantitative data were analyzed by ANOVA followed by Dunnett's multiple comparison tests. The Kruskal-Wallis test was used for the semiquantitative analysis of histopathology score values. Results were considered to be significant at *p* < 0.05.

## 3. Results

### 3.1. Blood/Serum Glycemic Parameters

**Figure 1:**
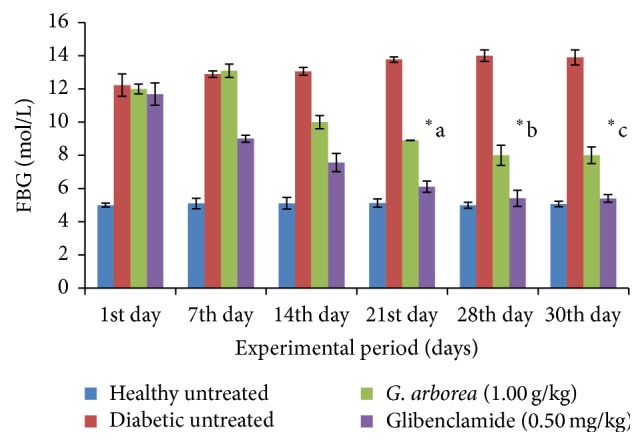
Effect of aqueous bark extract of* Gmelina arborea* (1.00 g/kg) fasting blood glucose concentration (FBG) in streptozotocin induced diabetic rats for 30 days. Data are expressed as mean ± SEM (*n* = 6/group). From the 21st day onwards, the fasting blood glucose concentration of* G. arborea* (1.00 g/kg) treated rats is significantly different from the fasting blood glucose concentration of diabetic untreated rats (^*∗*^a, b, c).

**Figure 2:**
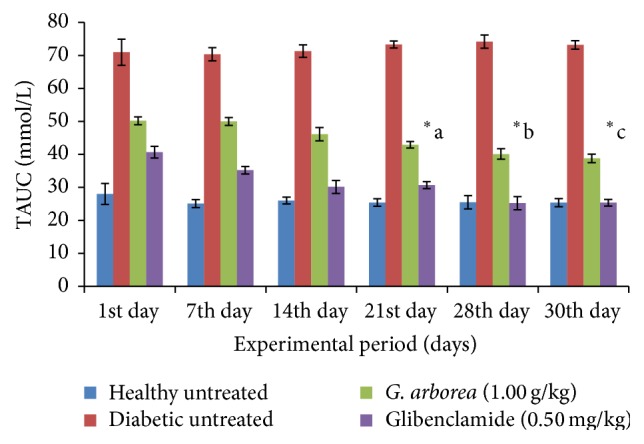
Effect of aqueous bark extract of* Gmelina arborea* (1.00 g/kg) on total area under the curve (TAUC) values in streptozotocin induced diabetic rats for 30 days. Data are expressed as mean ± SEM (*n* = 6/group). From the 21st day onwards, total area under the curve value of* G. arborea* (1.00 g/kg) treated rats is significantly different from the total area under the curve values of diabetic untreated rats (^*∗*^a, b, c).

Effect of the* G. arborea *(1.00 g/kg) extract on fasting blood glucose concentration in diabetic rats is shown in Figure 1. The healthy animals were normoglycemic throughout the experimental period. The fasting blood glucose concentration of* G. arborea *treated diabetic rats was reduced significantly from the 21st day onwards for the period of 30 days (*p* < 0.05). The reduction in fasting blood glucose concentration with the administration of* G. arborea *and glibenclamide was 37% and 42% in streptozotocin induced diabetic rats at the end of the study, respectively (*p* < 0.05). The total area under the curve values of plant extract treated diabetic rats showed a statistically significant improvement of 37% on the 30th day (*p* < 0.05, Figure 2). Effect of plant extract on the percentage of HbA_1C_ and concentration of fructosamine, insulin, and C-peptide in STZ diabetic rats is shown in Table 1. The diabetic rats treated with the plant extract exhibited a remarkable glycemic control as evident by a reduction in the percentage of HbA_1C_. The reduction in the percentage of HbA_1C_ and fructosamine was 31% and 28% in diabetic rats, respectively. However, glibenclamide treated diabetic rats demonstrated a fall of 40% and 43% in the above parameters in diabetic rats. The concentrations of serum insulin and C-peptide were increased significantly by 57% and 39% in plant extract treated diabetic rats, respectively (*p* < 0.05). The concentration of serum TC, HDL-C, LDL-C, VLDL-C, and TG in streptozotocin induced diabetic rats followed by the plant treatment is shown in Table 2. The streptozotocin induced diabetic control rats had a significant elevation in the concentration of serum TC (57%), LDL-C (93%), VLDL-C (95%), and TG (94%) and a reduction in HDL-C (12%) as compared with the untreated healthy control rats. The extract of* G. arborea *treated streptozotocin induced diabetic rats showed a significant reduction in the concentration of serum TC (31%), LDL-C (43%), VLDL-C (25%), and TG (29%) and an elevation in HDL-C (45%) on the 30th day of study (*p* < 0.05). The concentrations of serum TC, LDL-C, VLDL-C, and TG were reduced by 32%, 38%, 49%, and 48% in glibenclamide treated diabetic rats. In contrast, there was no significant change in the concentration of serum HDL-C with the glibenclamide treatment in diabetic rats (*p* > 0.05).

### 3.2. Histopathology and Immunohistochemistry of the Pancreas in Diabetic Rats

**Figure 3:**
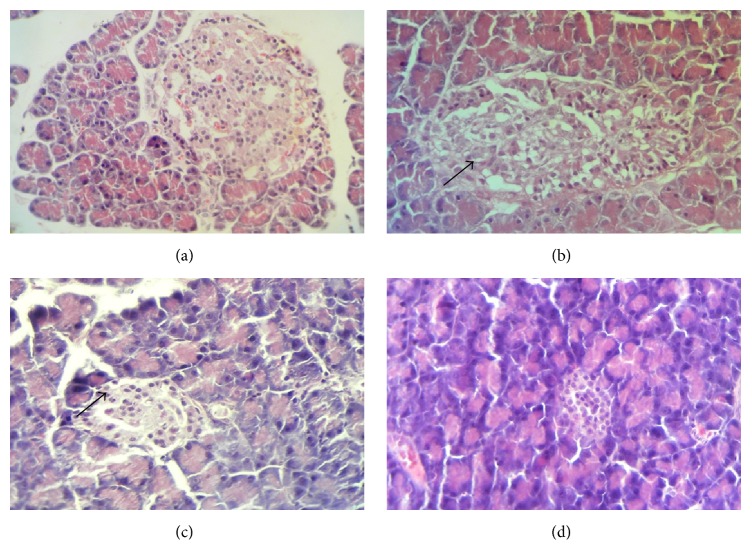
(a–d) Photomicrographs of histopathology of the pancreatic tissues, stained with hematoxylin and eosin (×400). (a) Healthy control rats, islets of Langerhans with normal islet cell population. (b) Diabetic control rats, an islet with few preserved islet cells, fibrosis, and infiltration by inflammatory cells. (c)* Gmelina arborea* (1.00 g/kg) treated diabetic rats, restoration of pancreatic islet cells with prominent islets. (d) Glibenclamide treated (0.50 mg/kg) diabetic rats with reduced number of islet cells.

**Figure 4:**
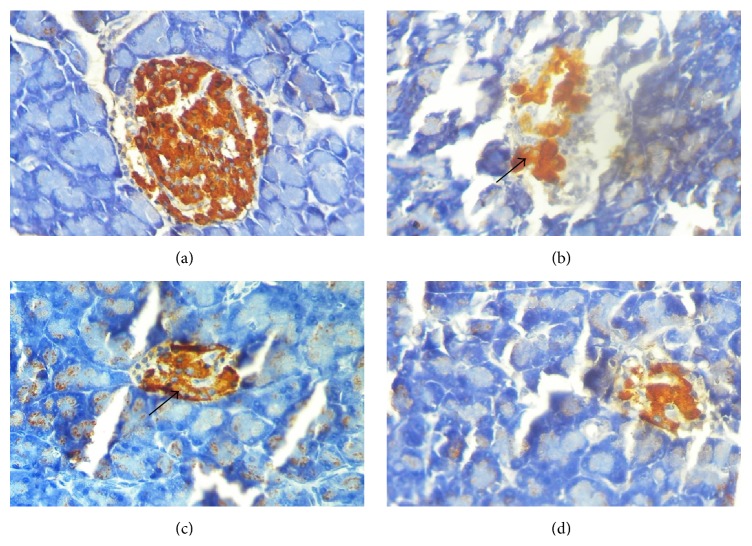
(a–d) Photomicrographs of insulin immune reactivity in pancreatic islets with anti-insulin antibody (×400). (a) Healthy control rats, a normal islet composed predominantly of insulin secreting cells. (b) Diabetic control rats, marked reduction in the number of insulin secreting *β*-cells due to the destruction of islet cells by streptozotocin. (c)* Gmelina arborea* (1.00 g/kg) treated (1.00 g/kg) rats, an islet with a marked increase in insulin secreting *β*-cells. (d) Glibenclamide treated (0.50 mg/kg) rats, mild increase in insulin secreting *β*-cells.

As shown in Table 3 and Figure 3, the sections of the pancreas from untreated diabetic rats showed an extensive destruction of islet cells as compared with that of healthy control rats (score value of 3 versus 0). Further, there was a definite reduction in the number of islets in diabetic rats, compared with that in the healthy rats. However, hemorrhages were not observed and acinar cells were intact in the pancreatic tissues of streptozotocin induced diabetic control rats. Further, severe inflammatory cell infiltrations in islets were also observed in diabetic control rats. Immunohistochemical staining with anti-insulin antibody confirmed a marked reduction (less than 10%) in insulin secreting cells in small, average, and large size islets in diabetic control rats (Table 4, Figure 4). The mean diameter of islets was reduced in small (15%), average (8%), and large (7%) islets in diabetic control rats as compared with the normal control rats. The sections from* G. arborea *extract treated diabetic rats revealed a statistically significant score value for the regeneration of islet cells with some hyperplastic islets as compared to diabetic untreated group (score value of 1 versus 0, *p* < 0.05). The number of islets was increased in plant treated diabetic rats when compared to diabetic control rats. Further the* G. arborea *extract produced a significant increase in the mean profile diameter in large islets (6%) as compared with the streptozotocin induced diabetic control rats.

## 4. Discussion

The present research was designed to evaluate antidiabetic effects of bark extract* G. arborea* (1.00 g/kg) in STZ diabetic rats. STZ diabetic rat is one of the most widely accepted models to investigate antidiabetic effects and mechanisms of action of any novel antidiabetic agents [26]. Streptozotocin (STZ) enters the pancreatic *β*-cells via a glucose transporter-GLUT2 and causes alkylation of DNA. The intraperitoneal injection of a single dose of STZ 65 mg/kg b. wt. exerts direct toxicity on *β*-cells resulting in necrosis within 48 hrs and causes permanent hyperglycemia. This causes the generation of superoxide, hydrogen peroxide, nitric oxide, and hydroxyl radicals which are responsible for *β*-cell damage and necrosis resulting in hyperglycemia [27–29].

Glibenclamide was used as the standard drug in the present study. It has been proposed that sulphonylureas exert antihyperglycemic effects through secretion of insulin from pancreatic *β*-cells and enhancement of insulin action on target tissues [30].

The study was performed for a period of 30 days in diabetic rats considering the life span of Wistar rats. In addition, many published studies on evaluating antidiabetic effects of medicinal plant extracts have been carried out in diabetic rats for a period of 30 days [11]. Diabetic animals showed an increase in blood glucose concentration which was reduced by the administration of* G. arborea *(1.00 g/kg). The antihyperglycemic activity was evident with the significant reduction of glycated hemoglobin in plant extract treated diabetic rats (*p* < 0.05). However, the reduction in the percentage of HbA_1C_ in glibenclamide treated diabetic rats was superior to the reduction in* G. arborea *extract treated diabetic rats at the end of the study period (30th day). The possible mechanism by which the extract produced the antidiabetic action in diabetic rats may be by potentiating the insulin effect of plasma by increasing the pancreatic secretion of insulin from the existing *β*-cells and promoting the regeneration of *β*-cells [31]. The pancreatic mechanism may be predominant because in mild diabetes mellitus induced by STZ all *β*-cells of the pancreas are not destroyed. Therefore, the surviving *β*-cells retain the capacity to synthesize and secrete insulin.

A significantly deranged lipoprotein profile was observed in STZ diabetic rats. This is in agreement with previous reports [32, 33]. The serum total cholesterol concentration was increased significantly in diabetic rats. Since insulin has a potent inhibitory effect on lipolysis in adipocytes, insulin deficiency is associated with excess lipolysis and increased influx of free fatty acids to the liver [34]. The increased levels of LDL-C and VLDL-C in diabetic rats might be due to overproduction of LDL-C and VLDL-C by the liver due to the stimulation of hepatic triglyceride synthesis as a result of free fatty acid influx. HDL-C was also significantly reduced in diabetic rats, which indicates a positive risk factor for atherosclerosis [35]. The concentrations of serum TC, TG, LDL-C, and VLDL-C were significantly reduced in the plant extract treated diabetic rats. This could be due to the reduction in hepatic triglyceride synthesis and/or lipolysis in plant extract treated diabetic rats [36]. Furthermore, the HDL-C was significantly increased in the plant extract treated animals indicating a reversed atherogenic risk (*p* < 0.05).

The optimum pancreatic *β*-cell function is essential for the regulation of intracellular glucose homeostasis. Several studies have provided evidence that loss of functional *β*-cell mass through apoptosis and impaired proliferation consequent to hyperglycemia is central to the development of both type 1 and type 2 diabetes mellitus. Indeed, it has been considered as a hallmark of both types of diabetes mellitus. Regulation of functional *β*-cell mass has been considered as a critical therapeutic challenge in patients with the disease. However, islet cell regeneration has gained much interest and has been considered as a strategy to restore the loss of *β*-cell mass in diabetes mellitus [36–38]. Insulin and C-peptide are the products of enzymatic cleavage of proinsulin and are secreted into the circulation in equimolar concentration. After insulin is released from the pancreas it undergoes significant first-pass clearance by the liver and therefore the estimation of serum insulin concentration alone may not be sufficient to confirm the insulin secreted by the pancreas. Therefore, estimation of both the concentrations of C-peptide and insulin has been reported to be valuable indices of insulin secretion [39]. The increment in serum insulin and C-peptide concentrations in plant extract treated rats corroborated the formation of functional islets and biosynthesis of insulin as evident through immunohistochemistry studies.

The results of the present study revealed a regeneration in the pancreatic *β*-cells in diabetic rats treated with the extract of* G. arborea *(1.00 g/kg). A pancreatic mechanism is possible because, in mild diabetes induced by STZ, all *β*-cells of the pancreas are not destroyed. The surviving *β*-cells retain the capacity to proliferate, synthesize, and secrete insulin [37]. The islet cell regeneration in diabetic rats with the plant treatments could be due to the replication of existing islet cells and differentiation (or neogenesis) from ductal or intraislet pancreatic precursor cells [38]. Various histopathological studies demonstrated an effective increase in the number of *β*-cells in the pancreas of diabetic rats treated with various plant extracts [39, 40]. The antidiabetic effects of the plant extracts are probably due to the presence of polyphenol compounds and flavonoids. Indeed, flavonoids as quercitrin and epicatechin are well documented for islet regeneration and enhancement of *β*-cell function* in vivo *[41].

## 5. Conclusions

The results confirm that the aqueous bark extract of* G. arborea* (1.00 g/kg) possesses* in vivo *antidiabetic activity through increased biosynthesis of insulin probably by *β*-cell regeneration in the pancreas of streptozotocin induced diabetic rats. In addition, the plant extract exerts antihyperlipidemic activities in diabetic rats. This is the first ever study to report the detailed pancreatic mechanisms of* G. arborea in vivo.* The findings of the present investigation revealed scrutinizing the therapeutic benefits of the* G. arborea *extract in the management of diabetes mellitus in traditional medicine.

## Figures and Tables

**Table 1:** Blood/serum glycemic parameters in diabetic rats after 30 days of treatment.

Treatment	Glycated hemoglobin (%)	Fructosamine (*µ*mol/L)	Insulin (*µ*IU/mL)	C-peptide (ng/mL)
Healthy untreated	4.86 ± 0.10	221.88 ± 3.10	14.23 ± 0.44	9.53 ± 0.80
Diabetic untreated	9.00 ± 0.09	405.39 ± 2.78	6.23 ± 0.09	5.75 ± 0.80
*G. arborea* (1.00 g/kg)	6.20 ± 0.06^*∗*^	292.30 ± 2.19^*∗*^	9.80 ± 0.10^*∗*^	7.98 ± 0.07^*∗*^
Glibenclamide (0.50 mg/kg)	5.38 ± 0.06^*∗*^	230.08 ± 0.99^*∗*^	11.75 ± 0.02^*∗*^	8.80 ± 0.01^*∗*^

The values are expressed as mean ± SEM (*n* = 6/group). ^*∗*^Statistically significant from streptozotocin induced diabetic control rats at *p* < 0.05 (ANOVA followed by Dunnett's test).

**Table 2:** Lipid profile in diabetic rats after 30 days of treatment.

Treatment	TC (mmol/L)	HDL-C (mmol/L)	LDL-C (mmol/L)	VLDL-C (mmol/L)	TG (mmol/L)
Healthy untreated	3.70 ± 0.08	1.25 ± 0.03	2.21 ± 0.02	0.22 ± 0.01	1.10 ± 0.09
Diabetic untreated	5.80 ± 0.05	1.10 ± 0.01	4.27 ± 0.18	0.43 ± 0.00	2.14 ± 0.05
*G. arborea* (1.00 g/kg)	4.00 ± 0.05^*∗*^	1.23 ± 0.01^*∗*^	2.47 ± 0.01^*∗*^	0.30 ± 0.03^*∗*^	1.50 ± 0.04^*∗*^
Glibenclamide (0.50 mg/kg)	3.95 ± 0.06^*∗*^	1.10 ± 0.09	2.63 ± 0.01^*∗*^	0.22 ± 0.03^*∗*^	1.12 ± 0.04^*∗*^

The values are expressed as mean ± SEM (*n* = 6/group). ^*∗*^Statistically significant from streptozotocin induced diabetic control rats at *p* < 0.05 (ANOVA followed by Dunnett's test). TC: total cholesterol; HDL-C: high density lipoprotein cholesterol; LDL-C: low density lipoprotein cholesterol; VLDL-C: very low density lipoprotein cholesterol; TG: triglyceride.

**Table 3:** Semiquantitative analysis of pancreatic tissue on selected parameters in streptozotocin induced diabetic rats after 30 days of plant treatment.

Treatment	Destruction of islet cells	Regeneration of islet cells
Healthy untreated	0	N/A
Diabetic untreated	3	0
*G. arborea* (1.00 g/kg)	0^*∗*^	1^*∗*^
Glibenclamide (0.50 mg/kg)	1^*∗*^	1^*∗*^

0: none; 1: mild; 2: moderate; 3: severe/prominent.

^*∗*^Statistically different from streptozotocin induced diabetic control rats at *p* < 0.05 (Kruskal-Wallis test).

**Table 4:** Effect of plant extracts on percentage of insulin secreting *β*-cells and diameter of islets in the pancreas of streptozotocin induced diabetic rats after 30 days of treatment.

Treatment	Percentage area of insulin secreting cells in islets (%)			Diameter of islets (*µ*m)		
	Small	Average	Large	Small	Average	Large
Healthy untreated	86.17 ± 3.54	72.00 ± 3.90	78.33 ± 7.53	86.80 ± 1.32	138.50 ± 5.57	173.16 ± 8.97
Diabetic untreated	9.17 ± 0.91	7.50 ± 1.23	6.83 ± 0.87	32.34 ± 1.55	127.43 ± 2.70	153.05 ± 0.37
*G. arborea* (1.00 g/kg)	60.89 ± 3.34^*∗*^	48.78 ± 4.78^*∗*^	69.00 ± 4.99^*∗*^	37.12 ± 2.13	137.00 ± 2.07^*∗*^	164.34 ± 2.56^*∗*^
Glibenclamide (0.50 mg/kg)	33.33 ± 2.34^*∗*^	10.00 ± 0.15^*∗*^	7.17 ± 1.42^*∗*^	36.10 ± 3.31	128.38 ± 1.99	154.08 ± 5.88

The values are expressed as mean ± SEM (*n* = 6/group). ^*∗*^Statistically significant from streptozotocin induced diabetic control rats at *p* < 0.05 (ANOVA followed by Dunnett's test).
