# Corrigendum to “*Gmelina arborea* Roxb. (Family: Verbenaceae) Extract Upregulates the *β*-Cell Regeneration in STZ Induced Diabetic Rats”

**DOI:** 10.1155/2018/9861243

**Published:** 2018-11-05

**Authors:** Anoja Priyadarshani Attanayake, Kamani Ayoma Perera Wijewardana Jayatilaka, Chitra Pathirana, Lakmini Kumari Boralugoda Mudduwa

**Affiliations:** ^1^Department of Biochemistry, Faculty of Medicine, University of Ruhuna, 80000 Galle, Sri Lanka; ^2^Department of Pathology, Faculty of Medicine, University of Ruhuna, 80000 Galle, Sri Lanka

In the article titled “*Gmelina arborea* Roxb. (Family: Verbenaceae) Extract Upregulates the *β*-Cell Regeneration in STZ Induced Diabetic Rats” [1], the following statement should be added as the second paragraph under the Animals subsection in the Materials and Methods:

Animals in the healthy control, diabetic control, and glibenclamide-treated groups overlap with another article titled “Antihyperglycaemic, Antihyperlipidaemic and *β* Cell Regenerative Effects of *Spondias pinnata* (Linn. f.) Kurz. Bark Extract on Streptozotocin Induced Diabetic Rats” [2], as the two studies were conducted in parallel by the same group. The use of common animals in control groups reduced the number of animals used in the experiments, an important aspect of the 3Rs principle in ethics involving animal research [3]. Furthermore, a common methodology was followed in the two studies [1, 2].
